# Preparation and characterization of myristic acid/expanded graphite composite phase change materials for thermal energy storage

**DOI:** 10.1038/s41598-020-67849-y

**Published:** 2020-07-02

**Authors:** Dongyi Zhou, Jiawei Yuan, Yuhong Zhou, Yicai Liu

**Affiliations:** 1 School of Mechanical and Energy Engineering, Shaoyang University, Shaoyang, 422000 China; 2 School of Energy Science and Engineering, Central South University, Changsha, 410083 China; 3 Key Laboratory of Hunan Province for Efficient Power System and Intelligent Manufacturing, Shaoyang University, Shaoyang, 422000 China

**Keywords:** Nanoscale materials, Energy storage, Thermoelectric devices and materials

## Abstract

Myristic acid/expanded graphite (MA/EG) composite phase-change material (CPCM) was prepared by absorbing liquid MA (as the PCM) into EG (as the supporting material). Its chemical structure, microstructure, and thermal properties were characterized and studied. In the MA/EG CPCM, the largest mass content of MA was 93.5% by using the diffusion–exudation circle method for the first time. Fourier transform infrared spectroscopy (FTIR) analysis indicated that the MA and EG were a pure physical mixture of which the structure does not change, and they undergo no chemical reaction. Differential-scanning-calorimetry (DSC) analysis results showed that the melting and freezing temperatures of the MA/EG CPCM were 53.3 and $52.4^{\,\circ }\hbox {C}$, respectively, and the melting and freezing latent heats were 189.5 and 187.8 J/g, respectively. After several heat-cycle accelerations, the material still had good thermal-energy-storage effect. MA/EG CPCM thermoconductivity was greatly enhanced after adding EG, and the results of thermal-storage/-release experiments indicated that the thermal-storage and -release ratios of the MA/EG phase-change unit was greatly improved when compared with that of MA. These results indicated that the MA/EG CPCM was a suitable low-temperature thermal-energy-storage material.

## Introduction

Because of the wide applications of thermal storage technology in many fields, such as solar-energy utilization, the recycling and utilization of industrial exhaust heat, peak-load shifting, and building heating ventilation and air conditioning, it has attracted increasing attention^1–4^. Thermal storage is generally classified into three methods, latent-heat thermal storage, sensible heat-thermal storage, and chemical-energy storage^5^. Of the three, latent-heat thermal storage has advantages such as stable temperature output, a distinct energy-saving effect, high-density energy storage, and wide use in solar-energy systems, including air conditioning, building-energy conservation, aerospace, and power systems^6–11^.

The core technology of latent-heat thermal storage is phase change materials (PCMs). Multiple inorganic, organic, and composite PCMs have been studied for building-energy efficiency, such as paraffin^12,13^, polyhydric alcohols^14,15^, inorganic salts^16^, and fatty acids^17^. In these materials, fatty acids are organic PCM that have attracted more attention due to their large latent heat, nontoxicicity, suitable transformation temperature, noncorrosiveness, low degree of supercooling, good thermal stability, and zero or minimal volume change^18,19^. The transformation temperature of fatty acids that are commonly used as phase-change energy-storage material ranges from 30.1 to $70.7^{\,\circ }\hbox {C}$, and its phase-change latent heat ranges from 149.1 to 222.8 J/g, which can be used for an energy-storage system of corresponding temperature^20,21^.

However, the thermoconductivity of fatty acids is relatively low, and leakage of the liquid phase occurs in the solid–liquid phase-change process, which severely restricts its application in thermal-energy storage. The most widely used method for improving the thermal-conductivity coefficient of fatty acid PCMs is the addition of a high-thermal-conductivity medium to obtain composite PCMs with good thermoconductivity. Substances that are generally used as a high thermal conductivity medium mainly include metal particles, metal oxides, and carbon materials (such as expansion ink and carbon nanotubes)^22,23^. Liquid-phase leakage can be avoided by encapsulating PCMs into stable composite PCMs in consideration of the inorganic porous medium as matrix. The final composite PCMs are prepared by absorbing fatty acid PCMs into their pore structure^24^. A porous matrix has a large specific surface area and multiple microporous structures in which the core material is distributed. The liquid-phase-change core material cannot easily overflow from the micropores due to the capillarity of micropores and the chemical bond force between the phase change core material and the matrix. Expanded graphite (EG) is widely used as a form-stable matrix because of its low density, high thermal conductivity, and multiple pores that can not only prevent liquid leakage, but also sharply enhance the thermal conductivity property of PCMs^25,26^.

Wang et al.^27^ prepared stearic acid/EG (SA/EG) composite PCMs with an SA mass content of 85%, and the corresponding phase-change temperature and latent heat were $128^{\,\circ }\hbox {C}$ and 187 J/g, respectively. Wu et al.^28^ prepared EG/SA composite PCMs, and found that the best condition was when SA mass content was 75% with packed density of 900$\,\mathrm{{kg/m}}^{3}$. Fang et al.^29^ prepared SA/EG form-stable PCMs with different mass rates, and found that, when EG content was 17%, the melting and freezing temperatures were 53.12 and $54.28^{\,\circ }\hbox {C}$, respectively. Moreover, the corresponding phase-change latent heats were 155.50 and 155.70 kJ/kg, and thermoconductivity increased almost 10 times compared to that of pure SA. SA in EG’s porous network was well-absorbed, and no SA leakage was found in the composite material, even in a molten state. Hu et al.^30^ prepared palmitic acid/EG (PA/EG) composite PCMs with an EG mass fraction of 1%, 3%, 5%, and 8%, and found that PA/EG composite PCMs preserve multiple pores of the EG material, the phase-change temperature of which is close to that of PA. Zhang et al.^31^ prepared CA–PA–SA/EG composite PCMs, of which EG quality content was 10%. Yang et al.^32^ prepared MA–PA–SA/EG composite PCMs, and found that PCM thermoconductivity was improved for the high thermoconductivity of the EG. Their research results proved that PCM thermoconductivity can be increased by adding EG.

MA, which melts at $53.6^{\,\circ } \hbox {C}$ with a latent heat of 199.4 kJ/kg and freezes at $51.8^{\,\circ } \hbox {C}$ with a latent heat of 199.0 kJ/kg, is beneficial organic PCM for thermal-energy storage. EG is a porous material with high thermoconductivity. However, studies of MA/EG composite PCMs for thermal-energy storage are rare. Cao et al.^33^ prepared MA/EG composite PCMs and confirmed that the best mass ratio of MA and EG was 15:1, and the transformation temperature and latent heat of MA/EG composite PCMs were $53.19^{\,\circ } \hbox {C}$ and 191.75 J/g, relatively, but some thermal properties have not been studied, such as thermoconductivity, thermal stability, and thermal reliability. Very little research has focused on the thermal properties of MA/EG composite PCMs. Hence, in this paper, MA/EG composite PCMs were prepared by absorbing liquid MA into EG. Their chemical structure, microstructure, and thermal properties were characterized and studied. The prepared MA/EG composite PCMs can be used for solar-energy-storage systems, that can store solar heat by daylight and release heat at night or on rainy days. This composite material can also be used for heat storage of a building exterio; it absorbs heat from its surroundings or solar radiation and releases it at night to reduce the load in air-conditioning systems.

## Experiments

### Materials

MA ($\geqq 98$ purity) as thermal-energy-storage material was supplied by Shanghai Zhunyun Chemical Co, Ltd., Shanghai, China. The grain fineness numb350 mesheser of the expandable graphite (Carbon content $>99$, expansion coefficient 100 mL/g, 350 meshes) was purchased from Qingdao Hengrunda Graphite Products Co, Ltd., Qingdao, China.

### MA/EG composite CPCM preparation

Expanded graphite was obtained by heating the dried expandable graphite in a porcelain crucible for 40 s in a $900^{\,\circ } \hbox {C}$ muffle furnace. A quantity of prepared expanded graphite and the MA were fully mixed in a beaker and then heated in boiling water that was constantly stirred until the MA melted. The melted MA was absorbed by EG. Then, the beaker was placed in a $100^{\,\circ } \hbox {C}$ vacuum-drying oven for dehydration. The mixture was stirred every 2 h until it cooled down to the indoor temperature to ensure the uniform mixing and absorption of MA and EG. Finally, MA/EG composite PCMs were obtained.

### MA/EG CPCM characterization

The transformation temperature (melting and freezing temperatures) and the phase-change latent heat (melting and freezing latent heat of the MA and the MA/EG eutectic mixtures before and after thermal cycling) were determined by differential scanning calorimetry (DSC; DSC instrument, STA2500, Germany) adjusted with inheatdium standard in a temperature range between 0 and $100^{\,\circ } \hbox {C}$. The growth ratio of DSC temperature measurement was $5^{\,\circ }\hbox {C/min}$, and the samples were cooled by liquid nitrogen. Same-sample DSC measurements were performed three times. The accuracy of phase-change temperature was $\pm 0.1\%$ and $\pm 4\%$ for latent heats. The extrapolated onset temperatures on the DSC curves were the transformation temperature of the PCMs.

The effect of the thermal-cycle-index on thermal properties was investigated by heating the MA/EG eutectic mixtures from a solid to a liquid state, and then cooling from a liquid to a solid state using a heat controller. When the numbers were 50, 100, and 200, the thermal cycling process was stopped. The property evolution of the MA/EG mixtures was surveyed by DSC, thermogravimetric analysis (TGA), and Fourier transform infrared spectroscopy (FTIR) techniques.

The thermal stability of the MA/EG was analyzed with TGA technique (TGA instrument, STA2500, Germany) with accuracy of $\pm 0.2\%$. The morphology and microstructure of the MA/EG composites were observed using a scanning electron microscope (SEM, S-4800, Hitachi Inc., Japan). Structural analysis of the MA/EG composites was conducted via FTIR (FTIR instrument, Thermo Scientific Nicolet iS5, USA). PCMs thermoconductivity was measured using a thermoconductivity analyzer (DRL-III, Xiangtan Xiangxi Instrument Co. LTD., China) with an accuracy of $\pm 3\%$.

## Results and discussion

### Maximal absorption ratio of MA in MA/EG CPCMs

Generally, the higher the mass of fatty acids in composite PCMs is, the better the thermal-energy storage of composite materials is. In the MA/EG composite PCM, EG was the carrier material in which MA was absorbed. When phase change occurs, MA changes from a solid state to a liquid state. However, due to the micropore capillarity and chemical-bond force between the phase-change core material and the matrix, MA will does not leak out of the composite PCM, and the composite PCM remains in its original form, i.e. a solid state. However, beyond the maximal absorption of the carrier materials, redundant fatty acids may attach to the surface of the composite PCMs, which can melt and lead to leakage during the phase-change process. EG content slightly affects the phase-transition temperature. Hence, the largest mass ratio of aliphatic acids in composite PCMs must be confirmed.

**Figure 1:**
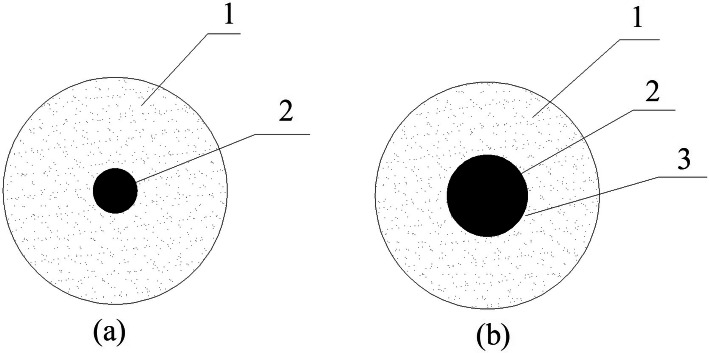
Diffusion–exudation-circle test diagram. (1. Filter paper; 2. composite material test area; 3. composite material exudation circle).

The diffusion–exudation circle method was applied to confirm the large mass rate of aliphatic acids in composite PCMs. The test diagram is shown in Fig. 1, and the test flow was as follows^34^. Composite PCMs samples were prepared with different MA contents (from 92 to 95%), and then equably dispersed in the test area with a diameter of 30 mm on the filter-paper center (as shown in Fig. 1a). Afterward, the specimens were moved to the vacuum drying-oven, heated at homeothermy of $70^{\, \circ } \hbox {C}$ for 30 min, and removed. The leakage degree of the phase change of the working substance component outside the self-tempering unit test area was observed (as shown in Fig. 1b). The largest and smallest diameters of the exudation circles that formed outside the exudation test areas were measured, and the average was calculated. Table 1 shows a series of regulated leakage percentages as the seepage-stability-assessment standard. The percentage of the excess of the average exudation-circle diameter to the test-area diameter was calculated, and the values were compared with the standard ones (Table 1) to assess stability. All values not higher than the standard indicated that stability reached the corresponding standards.

**Table 1:** Seepage-stability-assessment standard.

Item	Leakage percentage $\Phi$ (%)	Stability
Considered no exudation	$\Phi \le 10$	Very stable
Microscale exudation	$10< \Phi \le 15$	Stable
Slight exudation	$15< \Phi \le 30$	Basically stable
Medium amount of exudation	$30< \Phi \le 50$	Unstable
Large amount of exudation	$\Phi >50$	Extremely unstable

**Figure 2:**
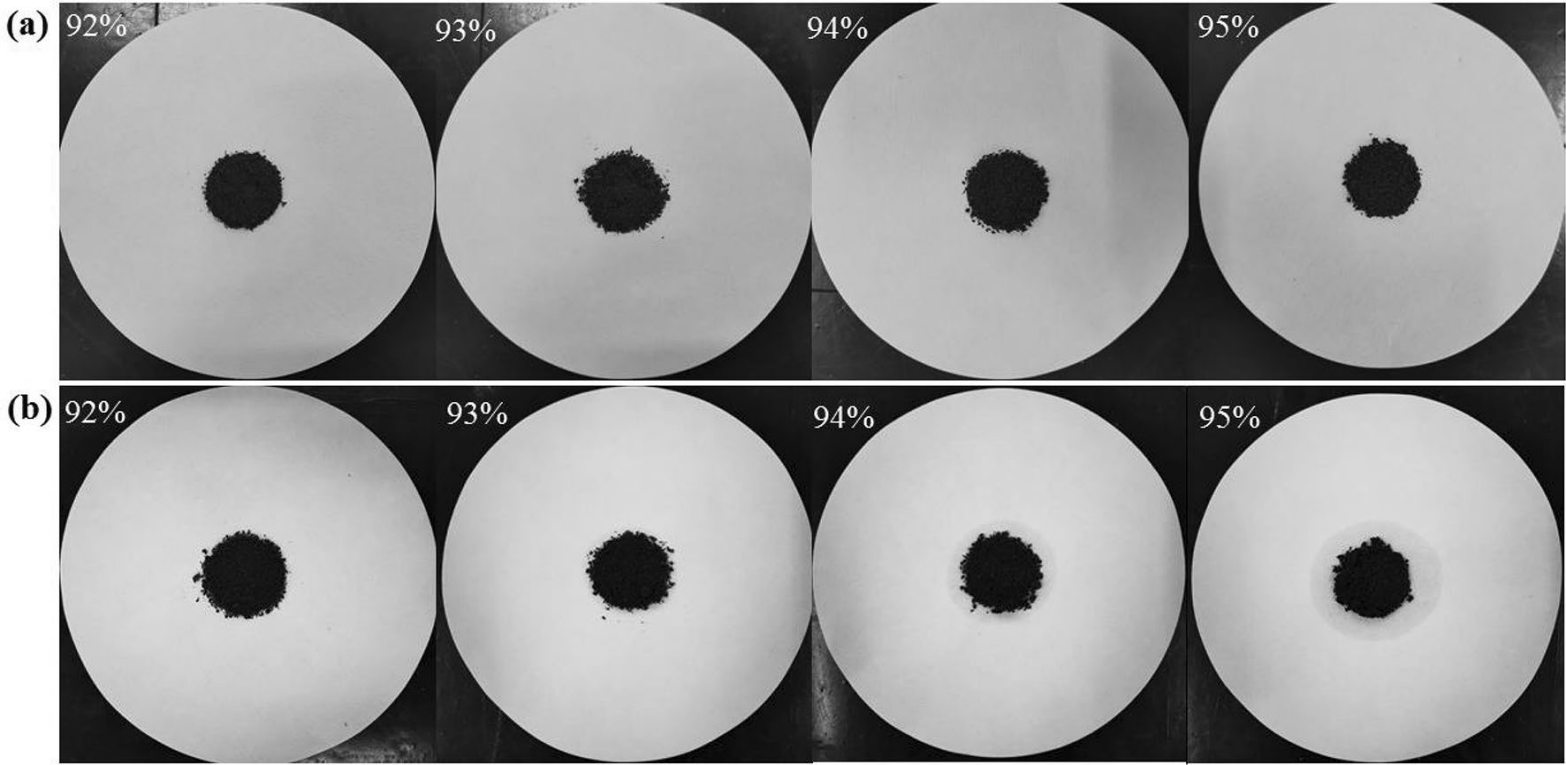
MA/EG CPCMs before (**a**) and after (**b**) heat treatment.

Figure 2 shows all photos of the prepared composite PCMs before (a) and after (b) testing. Leakage was evident in the sample with a 94% MA mass content, whereas no exudation was found in that with 93% MA mass content. Hence, the largest MA mass content was approximately 93–94%. Therefore, we prepared multiple samples with such mass content and applied the diffusion–exudation circle method to assess seepage stability; results are shown in Table 2. The largest MA mass content in the composite PCMs was approximately 93.5%. The MA/EG composite materials adopted all contents of MA at 93.5%.

**Table 2:** Fatty acid seepage-stability assessment.

MA quality content (%)	Average exudation-circle diameter (mm)	Leakage percentage $\Phi$ (%)	Assessment standard	Assessment result
92.0	35.34	17.8	$15{<}\Phi \le 30$	Basically stable
93.0	37.64	25.5	$15{<}\Phi \le 30$	Basically stable
93.2	37.85	26.2	$15{<}\Phi \le 30$	Basically stable
93.4	38.01	26.7	$15{<}\Phi \le 30$	Basically stable
93.5	38.14	27.1	$15{<}\Phi \le 30$	Basically stable
93.6	39.70	32.3	$30{<}\Phi \le 50$	Unstable
93.7	40.20	34.0	$30{<}\Phi \le 50$	Unstable
94.0	43.18	43.9	$30{<}\Phi \le 50$	Unstable
95.0	51.48	71.6	$\Phi {>}50$	Extremely unstable

### FTIR analysis of MA/EG CPCMs

**Figure 3:**
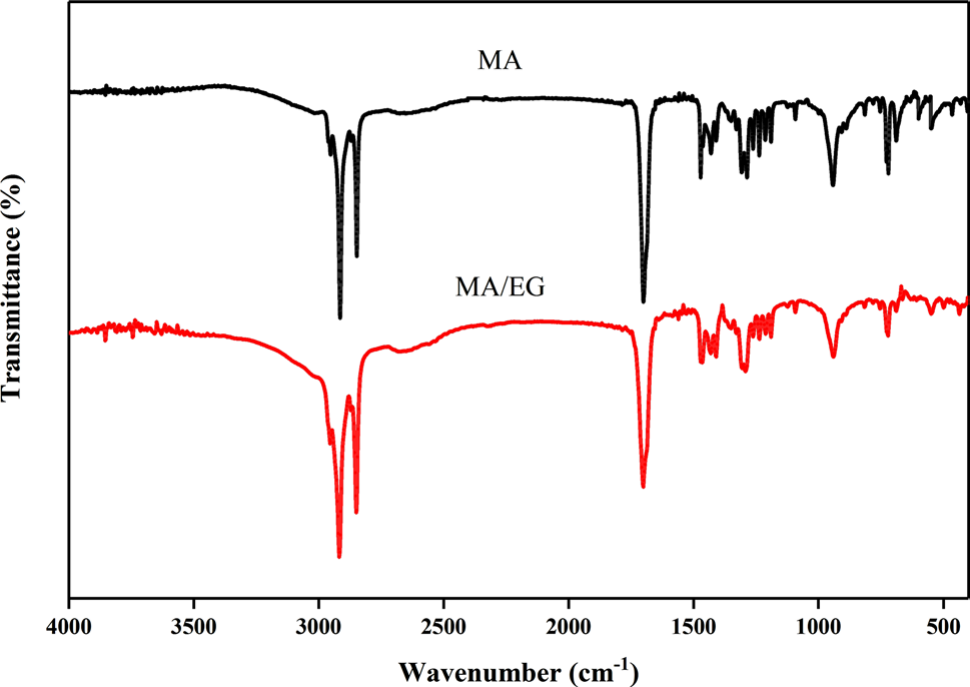
FTIR spectra of MA and MA/EG CPCMs.

The consistency and structural stability of the MA mixture with EG were investigated by conducting an FTIR test on pure MA and MA/EG composite materials; results are shown in Fig. 3. The infrared spectral curve showed a stretching vibration crest value at $1748 \, {\hbox {cm}}^{-1}$, of which the strong crest value was C=O on fatty acid carboxyl. Peaks occurred at 2945 and $2877 \, {\hbox {cm}}^{-1}$ due to the stretching vibration of the C–H bond in $-{\hbox {CH}}_{3}$ and $-{\hbox {CH}}_{3}^{-}$^35^. The spectra of MA and MA/EG were the same, and the positions of the characteristic peaks corresponded to one, indicating that the combination of MA and EG was a pure physical mixture. The structure did not change, indicating that no chemical reaction occurred.

### MA/EG CPCMs microstructure

**Figure 4:**
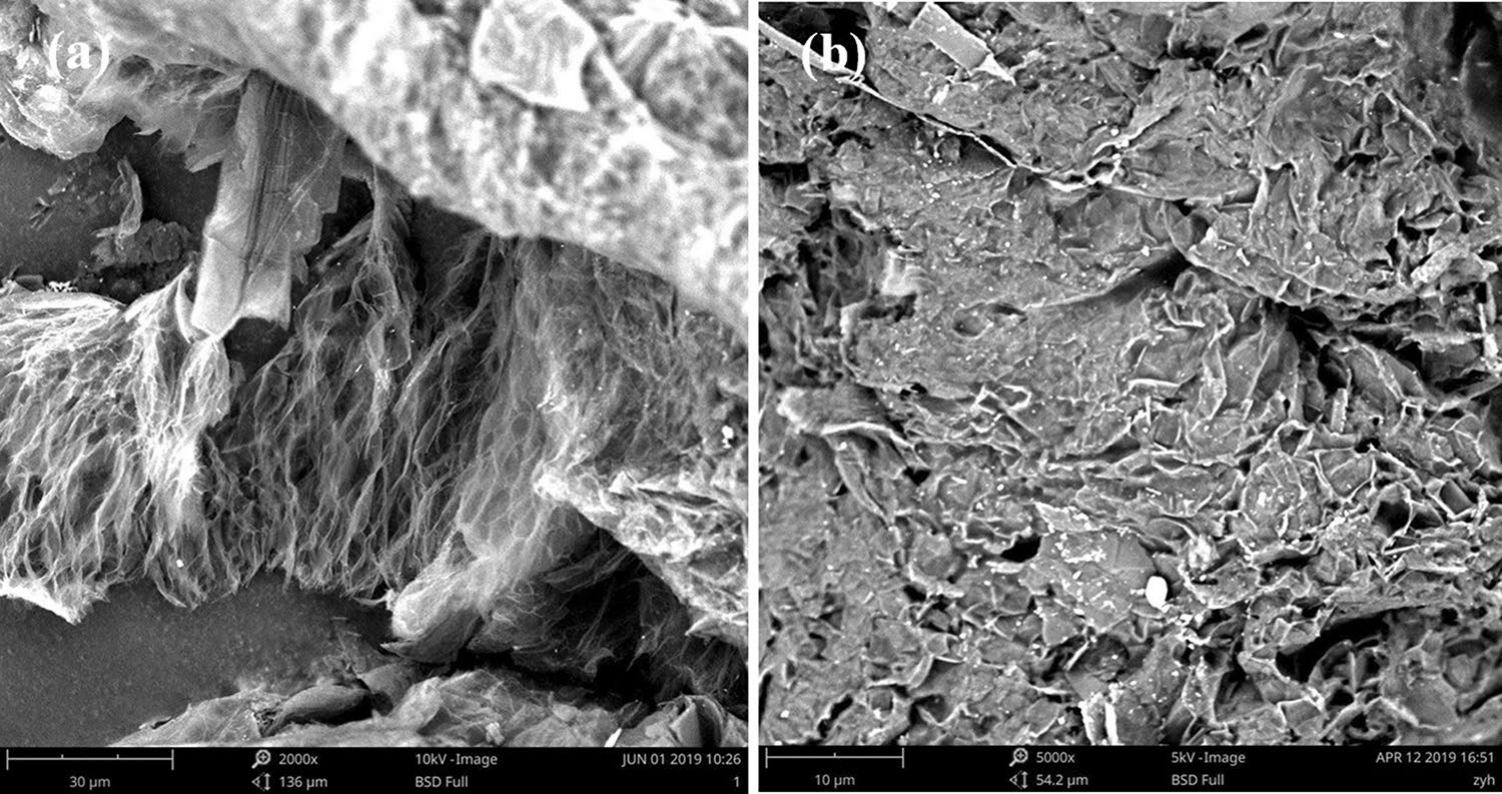
SEM pictures of EG (**a**) and MA/EG (**b**) CPCMs.

Figure 4 shows the microstructure of the EG and MA/EG composite materials, observed using SEM. The SEM picture of EG (Fig. 4a) shows that the EG pores were mainly at the microlevel, the wormlike porous structure caused EG to have a relatively large specific surface area and surface activity, and the molten MA was easier to absorb. In Fig. 4b, the SEM picture of MA/EG shows that MA was evenly dispersed in the porous structures of EG, in which the capillary tubes and surface tension could prevent the leakage of molten MA. On a macro level, the appearance of the MA/EG CPCM did not change during the phase change, indicating a certain form-stable effect. These observations revealed that, when the composite material was at the phase change, the molten MA in the pore network structure of EG had relatively good adhesive quality and compatibility, and MA was always equably distributed in EG without any leakage.

### MA/EG CPCM thermal properties

**Figure 5:**
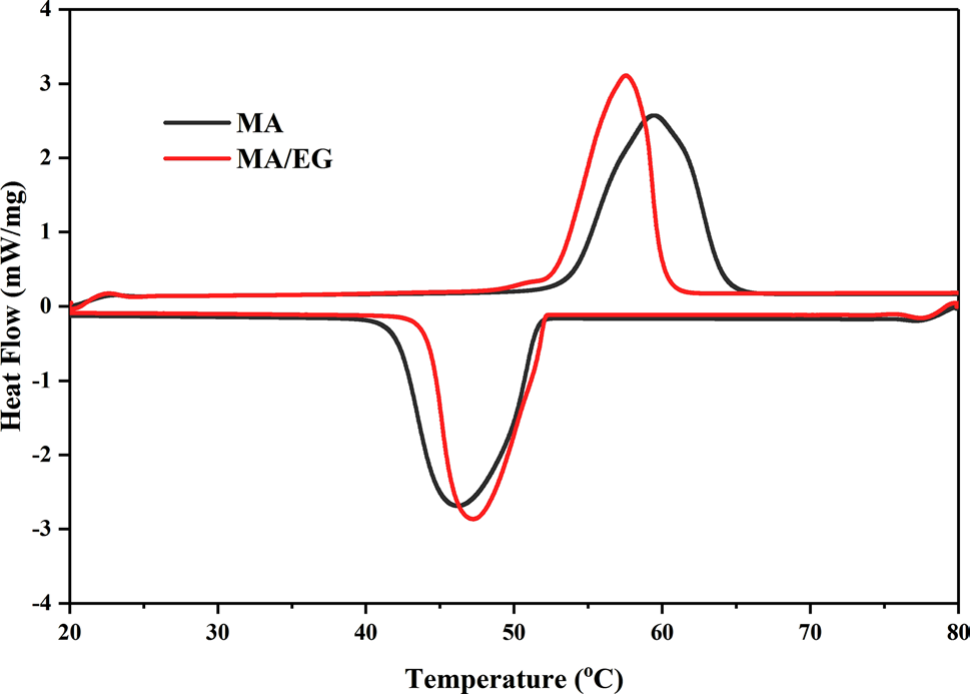
DSC curves of MA and MA/EG CPCMs.

The DSC curve of the MA/EG composite PCMs is shown in Fig. 5, and the thermal properties are shown in Table 3. As shown in Table 3, the phase-change characteristics of the MA/EG composites were very near those of the MA.

**Table 3:** DSC data of MA and MA/EG CPCMs.

PCM	Melting			Freezing		
	Onset temperature ($^{\circ } \hbox {C}$)	Peak temperature ($^{\circ } \hbox {C}$)	Latent heat (kJ/kg)	Onset temperature ($^{\circ } \hbox {C}$)	Peak temperature ($^{\circ } \hbox {C}$)	Latent heat (kJ/kg)
MA	53.6	59.5	199.4	51.8	46.3	199.0
MA/EG	53.3	58.0	189.5	52.4	48.1	187.8

Table 3 shows that the melting-point temperature of MA/EG was $53.3\;^{\circ } \hbox {C}$, and the freezing point temperature was $52.4\;^{\circ } \hbox {C}$. After a comparison, the melting temperature of the composite material was found to be slightly lower than that of MA, and freezing temperature slightly increased. However, the difference was small because MA was evenly dispersed in the mesh pore structure of EG, and EG has relatively high thermoconductivity. The high thermal-conductivity coefficient of the support material accelerated the heat-transfer rate of the PCMs from the external to the internal^36^, thereby resulting in lower phase-transition temperature.

Table 3 shows that the melting and freezing latent heat of MA/EG were 189.5 and 187.8 kJ/kg, respectively. Phase-change latent heat could also be calculated using Formula (1)^29^. Results are shown in Table 4. The difference between the experiment result and the calculation was less than 2%

1 $$\begin{aligned} \Delta H_{PCM}=\eta \Delta H_{AM} \end{aligned}$$

where $\Delta {\hbox {H}}_{PCM}$ is the calculated latent heat of the composite PCMs,$\eta$ is the mass percentage of MA in the composite PCM, and $\Delta {\hbox {H}}_{MA}$ is the latent heat of the MA as measured by the DSC.

**Table 4:** Comparison of experiment and calculated values of latent thermals of MA/EG.

MA mass content in MA/EG (%)	Melting			Freezing		
	Experimental value (kJ/kg)	Calculated value (kJ/kg)	Difference (%)	Experimental value (kJ/kg)	Calculated value (kJ/kg)	Difference (%)
93.5	189.5	186.8	1.5	187.8	186.3	0.8

The phase-change latent heat of the MA/EG was relatively lower than that of MA because in the MA/EG composite PCMs, EG only supported the internal MA, but did not influence the melt crystallization properties of the PCMs. Hence, the phase-change latent heat of the MA/EG CPCMs in the mass unit dwindled. The melting latent thermal energy of the MA/EG composite PCMs was reduced by 4.96%, and its freezing latent heat was reduced by 5.63%, which still had relatively high phase-change latent heat.

### Thermoconductivity measurement and thermal-storage/-release evaluation of MA/EG CPCMs

**Figure 6:**
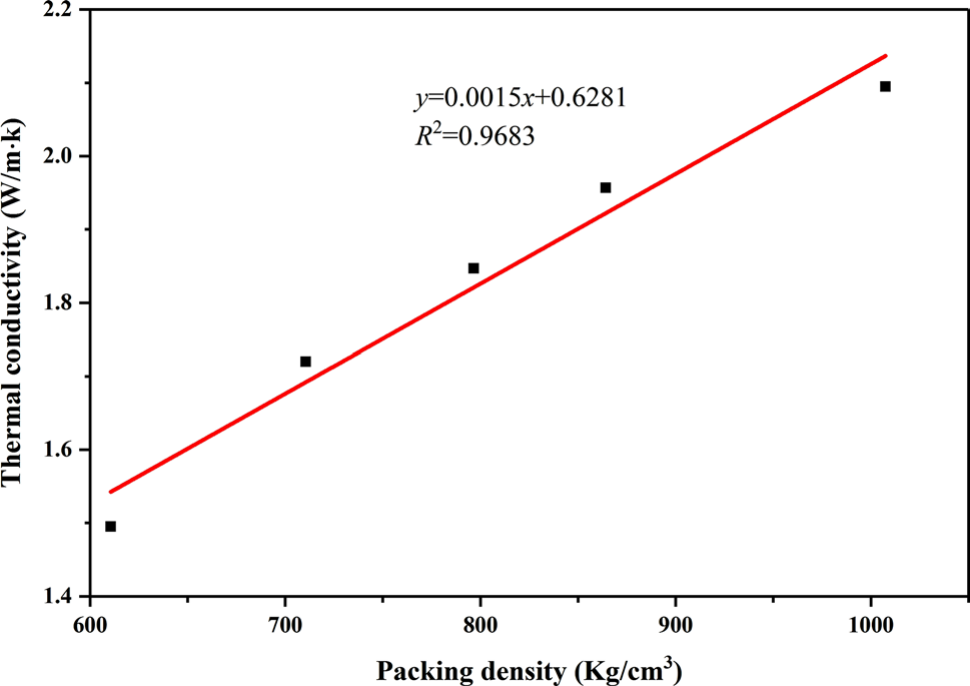
Thermoconductivity variation with packing density of MA/EG CPCMs.

MA belongs to organic PCMs with low thermoconductivit, and EG has relatively high thermoconductivity. Hence, the thermoconductivity of MA/EG composite material was greatly enhanced after adding EG. In this study, the MA/EG composite PCMs cylindrical blocks compressed with a smooth surface were formed by dry-pressing. Thermoconductivity-test data of the MA/EG composite PCM cylindrical samples with different densities are shown in Fig. 6. Results showed that the thermoconductivity of the samples increased with the increase of density. When sample density was 610.7, 710.5, 796.6, 864.2, and $1007.5 \, {\hbox {kg/m}}^{3}$, thermoconductivity was 1.495, 1.720, 1.847, 1.957, and 2.095 W/m K, respectively. Formula (2) was fitted from the data in Fig. 6 that showed a nearly linear relationship between the thermoconductivity (y) and packing density (x) of the MA/EG composite PCMs. The relation between thermoconductivity and packing density with different composite PCMs after adding EG can be found in relevant references^37,38^. This was probably caused by the extension of the contact surface area for the composite particles, and the reduction of the void space within the CPCM^39^. The higher the packing density of the CPCM was, the smaller the porosity in EG, the closer the contact among particles, the faster the heat-transfer speed, and the higher the thermal conductivity of the CPCMs were

2 $$\begin{aligned} y=0.0015x+0.6281\qquad (R^2=0.9683) \end{aligned}$$

**Figure 7:**
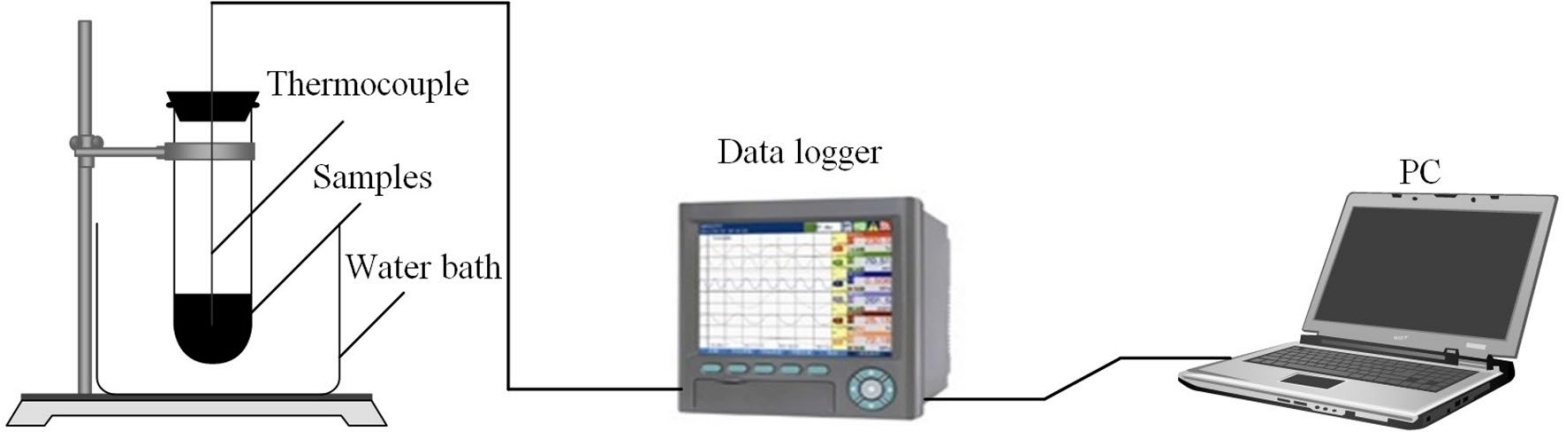
Experiment diagram for thermal-storage/-release measurement.

**Figure 8:**
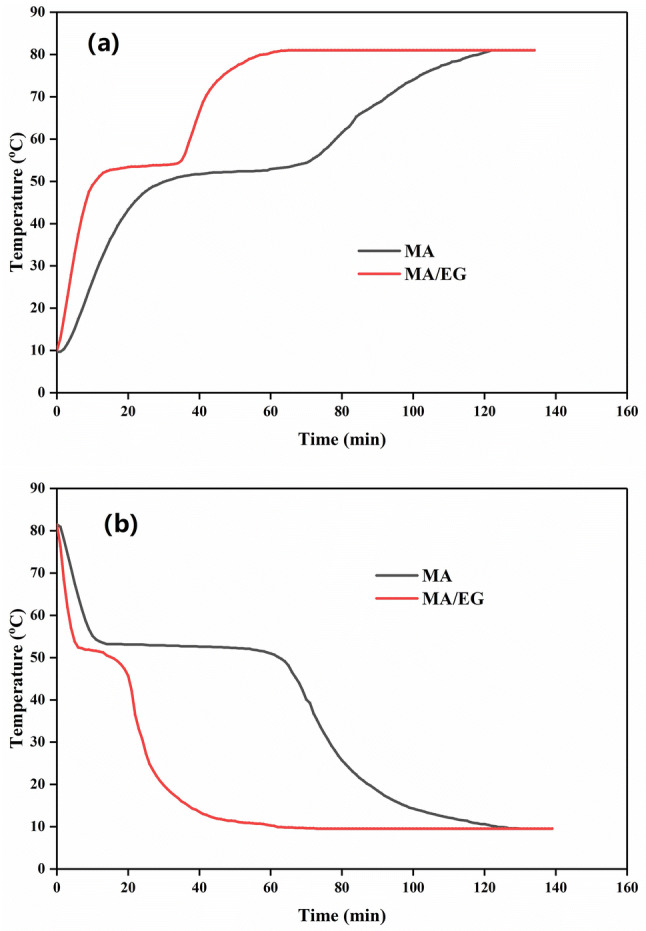
Storage (**a**) and release (**b**) curves of MA and MA/EG CPCMs.

EG addition enhanced the thermoconductivity of the composite PCMs. This finding could be proven by comparing the thermal-storage/-release characterization of MA and MA/EG composite PCMs. The test installation is shown in Fig. 7. The thermal-storage/-release rate could be assessed by the time when the temperature of the PCMs center reached the setting temperature during the phase-change processes. The temperature of the storage process was set $80^{\,\circ } \hbox {C}$, and that of the release process was set at $10^{\,\circ } \hbox {C}$. Results are shown in Fig. 8. Figure 8a shows the melting-temperature curves of the PCMs. The two curves indicate that the time required for heating MA/EG and MA from 10 to $80^{\,\circ } \hbox {C}$ was 58 and 118 min, respectively. The required time to complete the heat storage of MA/EG was 50.8% shorter than that of MA. Figure 8b shows the freezing-temperature curves of the PCMs, and indicating that the required time for cooling MA/EG and MA from 80 to $10^{\,\circ } \hbox {C}$ was 62 and 123 min, respectively. The required time to complete the heat release of MA/EG was 49.6% shorter than that of MA. Therefore, the thermal-storage/-release rate of the MA/EG composite PCMs was greatly improved when compared with that of MA because EG had a high thermal-conductivity coefficient. The thermal-conductivity coefficient of the composite PCMs was enhanced compared with that of MA, thereby accelerating the efficiency of heat transfer.

### MA/EG CPCM thermostability and seliability

**Figure 9:**
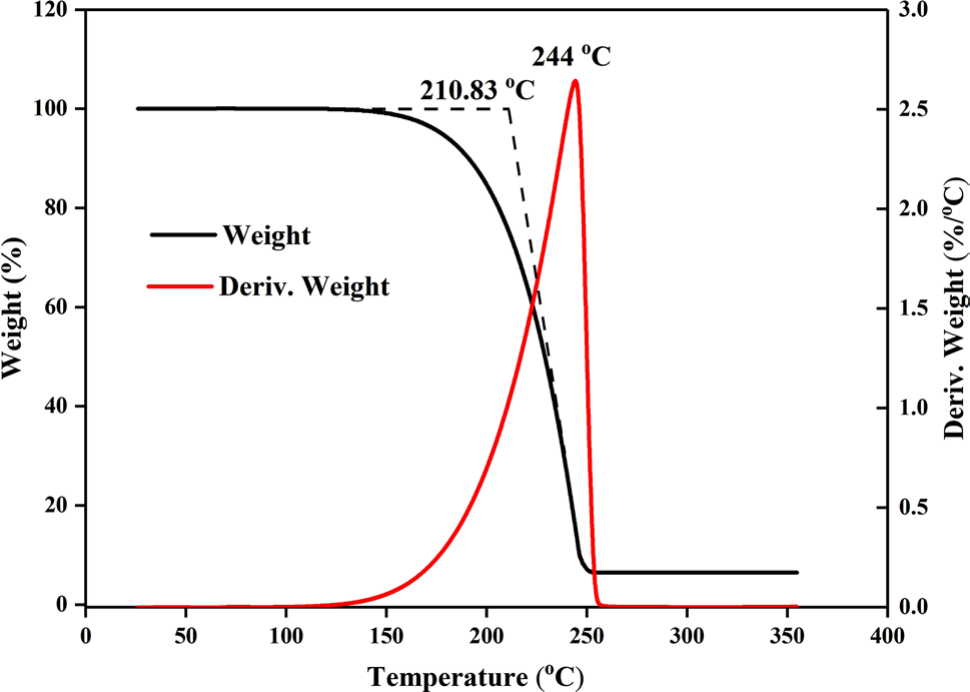
TGA curves of MA/EG CPCMs.

The thermostability of the MA/EG composite materials was assessed via TGA. The TGA curve is shown in Fig. 9. In the test, the MA/EG composite material was heated from indoor temperature to $80^{\,\circ } \hbox {C}$ and preserved for 60 min under such temperature with a constant weight-loss rate. Figure 9 shows that MA began losing weight from a temperature of approximately $120^{\,\circ } \hbox {C}$. Quality sharply decreased with increasing temperature. The weight-loss percentage reached the largest value at $210.83^{\,\circ } \hbox {C}$, and was almost completely volatilized at a temperature of approximately $244^{\,\circ } \hbox {C}$. This analysis indicated that, in a working environment of less than $120^{\,\circ } \hbox {C}$, even if the melting temperature of MA were surpassed, the MA/EG composite material would not lose MA. Hence, the MA/EG composite material has good heat stability in cryogenic applications of less than $100^{\,\circ } \hbox {C}$.

**Figure 10:**
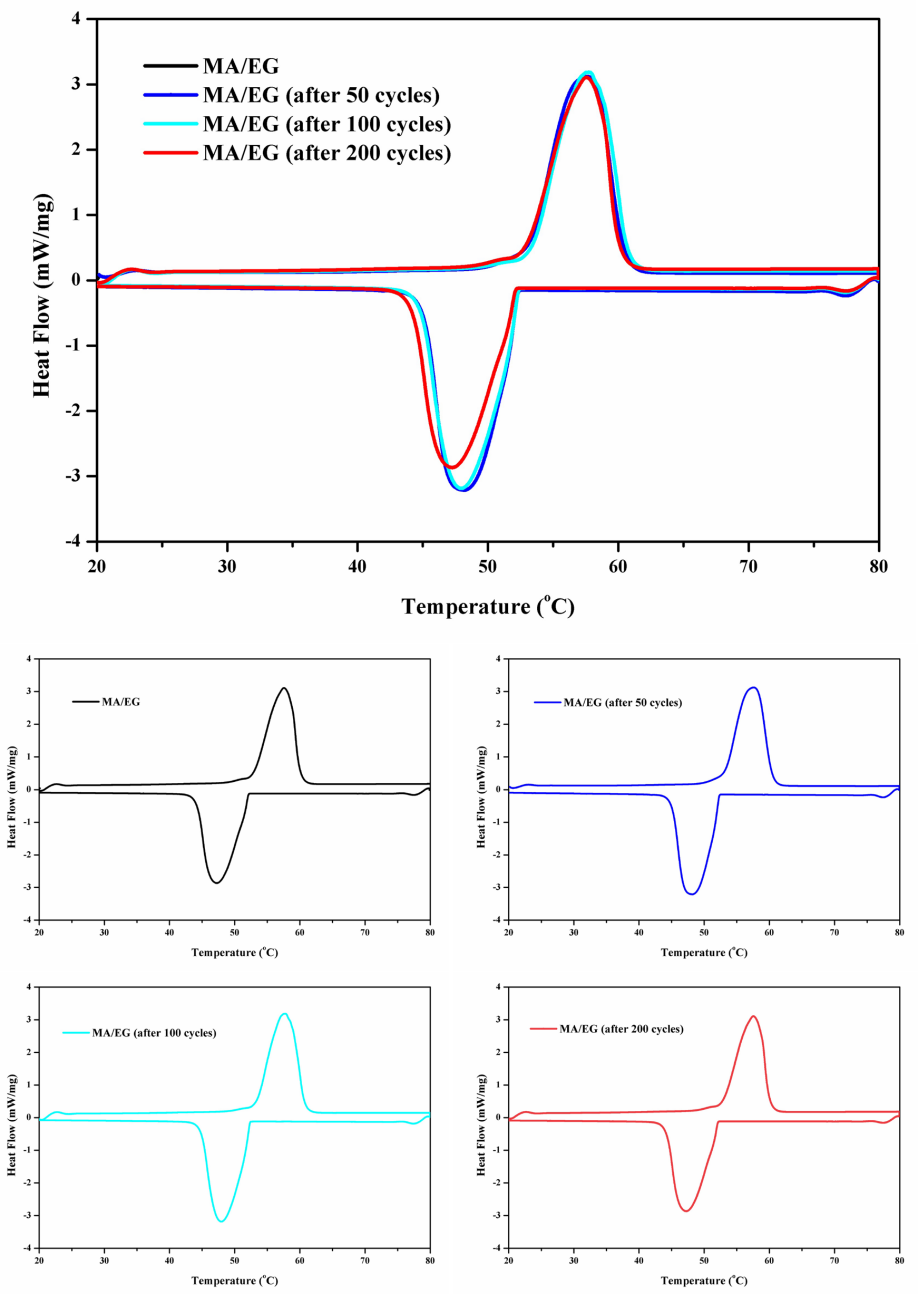
DSC curves of MA/EG CPCMs before and after thermal cycles.

The thermal-cycle reliability of PCMs means the attenuation of heat-storage performance after repeating heat-storage/-release processes. This parameter is important in evaluating the useful life of PCMs^40^. Thermal-cycle-acceleration experiment usually test for the thermal-cycle reliability of PCMs, which majorly concerns two important thermodynamic variables, namely, phase-transition temperature before and after a thermal cycle and phase-change latent heat. The DSC curves and heat performance, such as melting and freezing temperatures, and the latent heat of melting freezing of MA/EG after 50, 100, and 200 thermal cycles, are presented in Fig. 10 and Table 5. Figure 10 shows that the DSC curves of the MA/EG composite material before and after a thermal cycle were very close to each other. Table 5 shows that after 50, 100, and 200 times thermal cycles, the phase-change temperatures changed to 0.3, 0.1, and $0.5^{\,\circ } \hbox {C}$, and the phase-change latent heat changed to 0.1%, 0.9% and 4.5%. This outcome indicated that the MA/EG PCMs have good thermal-cycle reliability.

**Table 5:** Thermal performance of MA/EG CPCMs before and after thermal cycles.

Number of thermal cycling	Melting		Freezing		Extent of supercooling ($^{\circ } \hbox {C}$)
	Temperature ($^{\circ } \hbox {C}$)	Latent heat (kJ/kg)	Temperature ($^{\circ } \hbox {C}$)	Latent heat (kJ/kg)	
0	53.3	189.5	52.4	187.8	0.9
50	53.0	189.3	52.6	188.0	0.4
100	53.2	187.8	52.4	185.0	0.8
200	52.8	180.9	52.2	179.9	0.6

## Conclusion

In consideration of MA as PCM and EG as the carrier, and on the basis of the melt-adsorption method, an MA/EG composite phase-change energy-storage material was prepared with the largest MA quality content of 93.5%. FTIR test results indicated that no chemical reaction occurred before and after mixing MA and EG; EG only played the role of absorption, and a physical mixture was obtained. SEM characterization showed that MA could equably disperse in the EG network structure, and no seepage of liquid MA was found. DSC tests showed that the melting phase-change temperature and phase-change latent thermal energy of MA/EG PCMs were $53.3^{\,\circ } \hbox {C}$ and 189.5 J/g, and those of freezing were $52.4^{\,\circ } \hbox {C}$ and 187.8 J/g. The change of thermal properties was relatively smaller than that of pure MA. The TGA test proved that this phase-change energy-storage material has excellent stability within its operating temperature range. The thermal-cycle acceleration test showed that after 50, 100, and 200 cycles, the changes in the phase-change temperature and latent thermal energy of the ME/EG composite material could be neglected, and it has good reliability for long-term operation. The thermoconductivity of MA/EG CPCMs was greatly enhanced after adding EG, as verified by the thermal-storage/-release experiment. On the basis of this excellent performance, MA/EG composite PCMs can be widely used in low-temperature thermal-energy-storage systems, such as air-conditioning system, solar-energy storage, construction energy saving, and surplus-heat utilization.
